# The evolutionary safety of mutagenic drugs should be assessed before drug approval

**DOI:** 10.1371/journal.pbio.3002570

**Published:** 2024-03-15

**Authors:** Gabriela Lobinska, Vyacheslav Tretyachenko, Orna Dahan, Martin A. Nowak, Yitzhak Pilpel

**Affiliations:** 1 Department of Molecular Genetics, Weizmann Institute of Science, Rehovot, Israel; 2 Department of Mathematics & Department of Organismic and Evolutionary Biology, Harvard University, Cambridge, Massachusetts, United States of America

## Abstract

Some drugs increase the mutation rate of their target pathogen, raising the concern that they might thereby increase the pathogen’s rate of adaptation. This Perspective article proposes a four-step process to evaluate the evolutionary safety of new treatments, calling on regulatory authorities to take this into account before approval for widespread use.

## Teaser

Some drugs increase the mutation rate of their target pathogen, a potentially concerning mechanism as the pathogen might evolve faster toward an undesired phenotype. We suggest a four-step assessment of evolutionary safety for the approval of such treatments.

Mutations in pathogens are concerning. They can cause increased virulence or transmissibility, or resistance to therapy or vaccination. A drug that augments the mutation rate of a pathogen is a questionable proposition as its use could benefit the virus by enabling it to evolve faster, yet a growing number of pharmaceutical treatments increase the mutation rate of the viral or microbial pathogens they target [1,2]. If the increase in mutation rate is sufficient, the pathogen can acquire a lethal dosage of mutations, triggering an error catastrophe that prevents adaptation [3] or reduces the basic reproductive ratio to below one [4]. The concept of an error threshold is related to Muller’s ratchet or mutational meltdown [5,6]. Hence, the mechanism of action of mutagenic drugs is based on a population process. An example is the antiviral molnupiravir, an approved treatment for SARS-CoV-2 infection [7]. For other drugs, increased mutation rates are a byproduct of their intended effect, which relies on interfering with the replication process [8,9].

Mutagenic drugs are problematic, but resistance to antibiotics and antivirals present an urgent need for new drug development. We should thus explore every mechanism of drug action that has any chance of success. Yet, mutagenic drugs raise new safety concerns and require modified approval procedures. Clearly, any viral infection, even in absence of treatment, has the possibility to generate unwanted mutations. It is conceivable that by increasing the mutation rate of a virus, due to enhanced death rate, the total amount of unwanted mutants is reduced. In other words, untreated patients could generate more unwanted mutants than treated patients. Why is this the case? Increasing the mutation rate generates more lethal phenotypes, which harms the virus and helps the patient clear the infection faster. Therefore, patients that receive a mutagenic drug could have a lower total virus load and, consequently, a lower mutant virus load, integrated over the course of infection.

Below, we propose a scheme of 4 steps for evaluating the evolutionary safety of drugs. For each step, we provide suggestions for experiments, analyses, and tests to be performed (Box 1). Mutagenic drugs should be considered, but we think they need to be evaluated carefully for evolutionary safety, along with more common safety criteria, before they can be approved for use. We would define a mutagenic drug as being evolutionarily safe if it reduced the total amount of viable virus mutants produced during infection. Conversely, a treatment would be evolutionarily unsafe if it increased that quantity. A mutagenic treatment that is evolutionarily safe would be expected to reduce the rate of viral evolution in the population, although this concept has to be understood as a probabilistic statement. Of course, there could always be rare, unforeseen mutational events that lead to unexpected and dangerous variants. Any human intervention in evolutionary processes bears such a residual risk.

**Box 1. Suggested experiments for each step of the process for the evaluation of evolutionary safety**

**First step: Measurement of natural mutation rate, distribution of mutational effects, and virus infection dynamics**

- Measuring the natural mutation rate in cell-free systems, in cell culture, and by sequencing samples from patients.
- Establishing the number of lethal mutations and potential gain-of-function mutations through scanning mutagenesis of viral proteins to reveal amino acid positions that are susceptible to modify the function of the protein.
- Performing functional assays on potential mutants; for example, binding to antibodies or an entry receptor.
- Studying viral infection dynamics through repeated sampling of viral load during infection.

**Second step: Mutagenic potential of the drug**

- Sequencing and comparing the genomes of virus cultures from the treated and untreated samples to establish mutagenic potential and distribution of different types of mutations.
- Multiple passaging of the pathogen between cultures, with or without mutagenic treatment, to mimic evolution between individuals over several transmission events, then sequencing of the resulting strains to reveal the extent and nature of mutations.
- Direct testing of the resulting strains for undesired phenotypes, such as increased virulence, infectiousness, or resistance to treatment and immune evasion.
- Parallel repeats of the experiment to inform about the reproducibility of the evolutionary process under different treatment regimes.

**Third step: Preclinical and clinical trials in animals and human patients**

- Sampling of the genetic diversity of the viral population during the course of infection and compared between treated and untreated patients.
- Study of pharmacokinetics within the body.

**Fourth step: Surveillance after approval**

- Genomic analysis of sequences of the pathogen from epidemiological surveillance, looking for drug-specific signatures.

The first step will be to determine the genomic structure of the pathogen, derive a quantitative understanding of infection dynamics, and measure the natural mutation rate of the pathogen, including the recombination rate. All these quantities can affect the evaluation of evolutionary safety [10]. Of particular interest is the number of positions in the pathogen’s genome that are lethal to the pathogen when mutated, as the higher that number, the higher the likelihood for evolutionary safety [11].

The second step will consist of the study of the mutagenic potential of the drug. This step should apply both to drugs that are mutagenic by design and to drugs that exert mutagenesis as a by-product of their action. If there is mutagenic potential, then the magnitude of the effect must be evaluated in cell-free systems, treated bacteria, and viruses (as they infect cells in cultures). The mutation rate of the virus under treatment is dependent on drug dosage. Effective dosage in the body can be altered by poor treatment adherence, incomplete absorption, and fluctuations in drug concentration between doses. Therefore, the mutagenic potential should be established for a dynamic range of drug concentrations. Small increases in mutation rate are likely to reduce the evolutionary safety of a drug [10]. The treatment could be tested first in cell cultures: in a laboratory environment, a larger fraction of the pathogen’s genetic diversity could be observed than in vivo. The observed viral diversity in vivo depends on the sampling methods and on the pathogens’ distribution within the body. Different concentrations and regimes of the treatment could be tested, controlling for pharmacokinetic aspects in different parts of the body.

In the third step, the assessment of evolutionary safety would proceed into preclinical and clinical trials. Evolution experiments that were performed in the second step, involving taking the pathogen through serial passaging in cell culture, could be repeated in appropriate animal models that are infected with the pathogen and treated with the drug. Pharmacokinetics of the mutagenic treatment within the body could result in different mutation rates than those measured in vitro. Therefore, the viral mutation rate in treated animals or human patients has to be measured, taking into account factors such as poor treatment adherence or excluded sites in the body where the pathogen can replicate but the drug cannot fully reach. Evolutionary safety can be demonstrated if treatment results in a lower cumulative mutant pathogen load. Evolutionary safety could depend on the patient’s immune response and other parameters [10]. It is conceivable that a drug could be deemed evolutionarily safe in some patients groups but unsafe in others.

The fourth step would occur after the drug had been approved and population-wide prescription of the drug ensued. Characteristic mutational patterns could then be linked to mutagenic treatments. For example, the mutational signature of molnupiravir was detected in sequences of SARS-CoV-2 from countries where it was widely used [8], but the study did not reveal if there was an increase in viral diversity as a consequence of treatment with the drug. The appearance of mutational signatures caused by mutagenic treatment does not in itself exclude the possibility that a drug is evolutionarily safe. However, it should be the case that evolutionarily safe treatments reduce the number of new infections and the total amount of virus mutants present in a population. A goal of evolutionary safety assessment will also be to reveal drug administration regimens and pharmaceutical development directions that maximize evolutionary safety. Lack of treatment adherence may affect evolutionary safety; hence, mutagenic drugs might be restricted to hospital use.

Lethal mutagenesis is a relatively new and potentially important strategy for the treatment of infections. The recent SARS-CoV-2 pandemic has underscored the importance of the production of new antiviral drugs. However, evolutionary safety is not confined to the treatment of SARS-CoV-2, it can be applied to other viruses and multidrug-resistant bacterial strains. For example, phage therapy [9], although promising, raises an evolutionary safety concern as it may lead to unpredictable evolutionary outcomes such as increased target resistance towards both phage and antibiotics [12]. A consistent procedure to assess the evolutionary safety of such mutagenesis treatments would allow us to use them without an otherwise significant risk to humanity. Evolutionary safety also has moral and ethical implications. A potential ethical dilemma could occur if, for example, a higher dose of a drug presents more toxicity or adverse effects but is necessary for increased evolutionary safety.

We urge regulatory bodies to consider incorporating evolutionary safety approval to the authorization process for new drugs. We call upon pharmaceutical companies and researchers to include evolutionary safety in their R&D considerations. We also urge clinicians to become aware of potential effects of prescription and administration regimes on evolutionary safety for the benefit of humanity.
